# Advancements in the Research on the Preparation of Isoamyl Acetate Catalyzed by Immobilized Lipase

**DOI:** 10.3390/ma18112476

**Published:** 2025-05-25

**Authors:** Guoqiang Guan, Yuyang Zhang, Jingya Qian, Feng Wang, Liang Qu, Bin Zou

**Affiliations:** 1 School of Food and Biological Engineering, Jiangsu University, No. 301 Xuefu Road, Zhenjiang 212013, China; ggqyxq@ujs.edu.cn (G.G.); 2212218043@stmail.ujs.edu.cn (Y.Z.); qianjingya@ujs.edu.cn (J.Q.); fengwang@ujs.edu.cn (F.W.); 2 School of Food and Biological Engineering, Wuhu Institute of Technology, Wuhu 241003, China; 101385@whit.edu.cn

**Keywords:** immobilized lipase, isoamyl acetate, biocatalysis, solvent engineering

## Abstract

This study aims to delve into the application potential of immobilized lipases in the catalytic synthesis of isoamyl acetate. Through a comparative analysis of various immobilization methods, including physical adsorption, encapsulation, covalent binding, and crosslinking, along with the utilization of nanomaterials, such as magnetic nanoparticles, mesoporous silica SBA-15, and covalent organic frameworks (COFs) as carriers, the study systematically evaluates their enhancing effects on lipase catalytic performance. Additionally, solvent engineering strategies, encompassing the introduction of organic solvents, supercritical fluids, ionic liquids, and deep eutectic solvents, are employed to intensify the enzymatic catalytic process. These approaches effectively improve mass transfer efficiency, activate enzyme molecules, and safeguard enzyme structural stability, thereby significantly elevating the synthesis efficiency and yield of isoamyl acetate. Consequently, this research provides solid scientific rationale and technical support for the industrial production of flavor ester compounds.

## 1. Introduction

Flavor esters, as important aroma compounds in food additives, are primarily synthesized through esterification reactions and are widely used in the beverage, baking, confectionery, and other food industries [1,2]. Based on their aroma characteristics, they can be classified into three major categories, as follows: fruity aroma type (such as isoamyl acetate and ethyl butyrate), floral aroma type (such as methyl benzoate and geranyl butyrate), and mixed aroma type [3]. Among these, esters with a fruity aroma account for over 65% of the market share [4].

Isoamyl acetate, with the chemical formula C_7_H_14_O_2_, is a naturally occurring ester compound characterized by a unique aroma. It typically appears as a colorless to pale yellow transparent liquid, emitting a combined fragrance reminiscent of bananas and pears [5,6]. In the food industry, it serves primarily as a food additive in beverages, candies, and dairy products, enhancing or imparting a desirable fruity flavor. Distinguished from other similar products, its volatile properties align closely with the sensitive range of human olfaction, allowing for significant flavor enhancement at low concentrations [7]. Industrially, the synthesis of this ester utilizes isoamyl alcohol and acetic acid, which have a stable supply chain, and the reaction conditions are mild (60–80 °C), making it more economically viable compared to similar products, such as ethyl butyrate, which require high temperatures and pressures [8,9]. According to global fragrance market reports, isoamyl acetate has consistently ranked among the top three best-selling food flavorings for five consecutive years, with an application coverage rate of 78% in the soft drink sector. This is closely related to its stability within the pH range of 3–8, enabling it to adapt to the processing requirements of various food matrices [10,11].

In the food industry, isoamyl acetate is extensively employed for the formulation of various fruit-flavored edible flavorings due to its distinctive aroma. Whether it is for banana, apple, strawberry, grape, or pineapple flavor profiles, isoamyl acetate can impart a rich and authentic fruity aroma, enhancing the flavor of food products. Furthermore, it is permitted for use as an edible flavoring agent to ensure the taste and safety of food [12,13]. Yin et al. optimized process parameters to control the content ratio of ethyl acetate to isoamyl acetate in the final beer product within the range of 5.9:1 to 6.1:1, thereby conferring the beer with its characteristic taste and aroma [14]. Wang et al. introduced sterilized isoamyl acetate into a selective culture medium and, through subsequent isolation and purification, obtained isoamyl acetate-producing strains, providing valuable insights for the development and utilization of flavor-enhancing strains in fermented foods, such as alcoholic beverages and soy sauce [15].

Given that isoamyl acetate, a naturally occurring ester compound with a unique fragrance blending the aroma of bananas and pears, holds significant potential for widespread application as a crucial food additive in the food industry, this review aims to comprehensively summarize the latest research advancements in the field of the immobilized lipase-catalyzed synthesis of isoamyl acetate. Through an in-depth analysis of the impact of various immobilization methods and carrier materials on the catalytic performance of lipase, this study not only elucidates how immobilization technology markedly enhances the stability, reusability, and catalytic efficiency of lipase but also explores the potential and advantages of this technology in enabling large-scale production of flavor esters, such as isoamyl acetate. This review innovatively compiles recent application examples of immobilized lipase in the synthesis of isoamyl acetate, systematically compares the merits and demerits of different immobilization methods, and envisions the innovative application of solvent engineering in intensifying the enzymatic catalytic process. It provides a comprehensive theoretical foundation and technical support for the industrial production of isoamyl acetate, which is of great significance for advancing the green development of the food industry.

## 2. Preparation Method of Isoamyl Acetate

**Figure 1:**
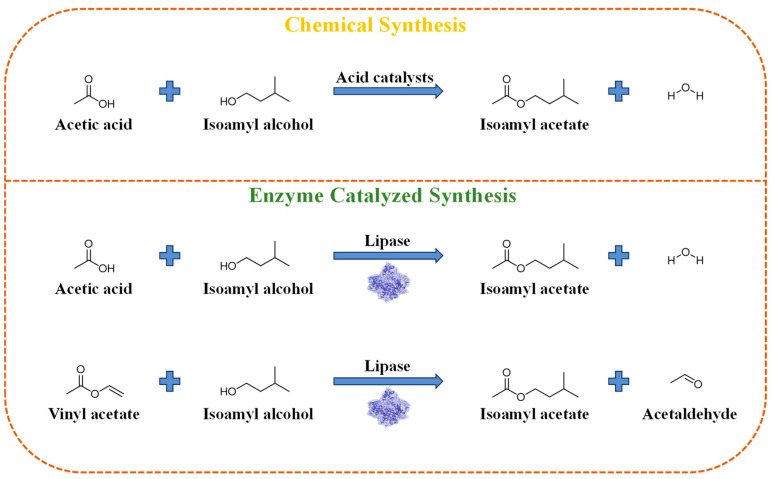
Synthesis method of isoamyl acetate.

Isoamyl acetate can be obtained through natural extraction, as well as prepared via organic synthesis and enzymatic catalysis (Figure 1). Isoamyl acetate is widely found in nature. It is abundant in fruits, such as bananas, apples and strawberries. In theory, it can be separated from natural substances containing this component using extraction methods. However, this approach often faces challenges, such as low extraction efficiency, insufficient purity, and high costs [16]. Moreover, due to the limited content of naturally derived isoamyl acetate, its current primary application is in high-value cosmetics. This method is insufficient to meet the demand for large-scale production of food flavor esters [17].

Chemical synthesis, utilizing acetic acid and isoamyl alcohol as raw materials and catalyzed by acidic catalysts, is employed for the esterification to produce isoamyl acetate. However, this method is not deemed optimal for the production of isoamyl acetate intended for use as a food additive due to its inherent limitations. In contrast, enzymatic synthesis, as an emerging technology, has garnered considerable attention owing to its environmentally friendly nature, high efficiency, energy conservation, and mild reaction conditions.

The chemical synthesis method involves the esterification of acetic acid with isoamyl alcohol as substrates, catalyzed to produce isoamyl acetate. This reaction typically proceeds under acidic conditions, with commonly used catalysts including inorganic acids, such as sulfuric acid and phosphoric acid, as well as solid acids, like ion exchange resins [18]. Wang et al. employed ion exchange resin-supported (NH_4_)_6_[MnMo_9_O_32_]·8H_2_O with a Waugh structure to prepare a supported solid catalyst. Under optimized reaction conditions, the yield of isoamyl acetate reached 95.1% [19]. Furthermore, the esterification reaction is reversible, and to enhance the yield, measures are often taken to drive the reaction towards ester formation. These measures include adding an excess of isoamyl alcohol substrate, lowering the reaction temperature, and promptly removing the generated water [20,21]. Mansor et al. added an excess of alcohol in the synthesis of isoamyl acetate, providing a high tendency for the reaction to proceed in the forward direction and ultimately increasing the ester yield [22]. The advantages of the chemical synthesis method include the easy availability of raw materials, rapid reaction rates, and low catalyst costs [23]. However, this method suffers from drawbacks, such as environmental pollution caused by acidic catalysts and the production of numerous high-temperature reaction byproducts, making it difficult to obtain high-quality products. Additionally, considering food safety, it is not the optimal production process for isoamyl acetate intended as a food additive [24].

Enzymatic synthesis has emerged as a novel technique for the preparation of isoamyl acetate, garnering considerable attention due to its green, efficient, and mild-condition advantages [25]. The interfacial activation of lipase at the oil–water interface can maintain the stability of the transition intermediate products, so as to realize an efficient catalytic esterification reaction. This method involves the esterification of acetic acid and isoamyl alcohol, catalyzed by enzymes under mild conditions to produce isoamyl acetate. Narwal et al. immobilized lipase from *Bacillus aerius* onto a silica gel matrix using a crosslinker, glutaraldehyde, and investigated its efficiency in catalyzing the esterification reaction for the synthesis of isoamyl acetate, achieving a yield of 68% under optimal reaction conditions [26]. Dos Santos et al. employed the commercial enzyme Novozyme 435 to catalyze the esterification of acetic anhydride and isoamyl alcohol in both batch and packed-bed reactors, demonstrating that the packed-bed reactor yielded a higher isoamyl acetate production rate than the batch reactor [27]. Zare et al. utilized Novozyme 435 to catalyze the esterification of acetic anhydride and isoamyl alcohol, enhanced by microwave irradiation (100 W) during the reaction, achieving a 100% yield of isoamyl acetate within 1 h [28]. Despite the significant progress made in current enzymatic synthesis processes, there remain shortcomings, such as low lipase activity, prolonged reaction times, and lower product yields [29,30].

## 3. Research Progress on Enzymatic Synthesis of Isoamyl Acetate

### 3.1. Overview of Lipase

**Figure 2:**
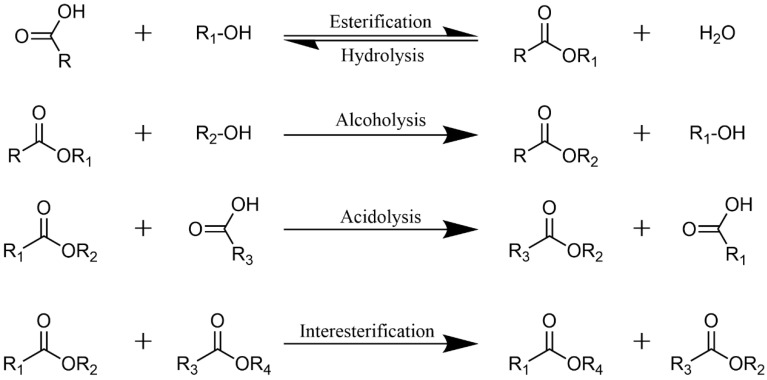
Types of lipase-catalyzed reactions.

Lipase (EC 3.1.1.3) is ubiquitously present in the tissues of animals, plants, and microorganisms (such as molds and bacteria). It serves as a biocatalyst for various reactions, including esterification, transesterification, acidolysis, and alcoholysis (Figure 2). Due to its high efficiency, selectivity, mild catalytic conditions, and relatively high enzymatic activity, lipase exhibits broad application potential [31,32]. Among the microbial sources of lipases, yeast, as a eukaryotic organism, often secretes lipases with more complex glycosylation modifications, which subsequently influence the stability and functional properties of the enzyme. Among them, *Candida rugosa* has garnered significant attention due to its ability to secrete multiple isozymes, with its lipase maintaining high activity in organic solvents. *Candida utilis* predominantly secretes extracellular lipases, making it suitable for large-scale fermentation production. The *Pichia* species has emerged as a popular host for recombinant lipase production owing to its ease of genetic manipulation and high expression levels. Lipases from the *Rhodotorula* species and *Yarrowia* species exhibit application potential in specialized industrial scenarios due to their adaptability to extreme environments, such as high temperatures and high salinity. Through genetic engineering modifications or fermentation process optimization, these yeast strains can further enhance lipase yield and performance, thereby providing crucial technological support for green biomanufacturing [33].

### 3.2. The Catalytic Mechanism of Lipase

**Figure 3:**
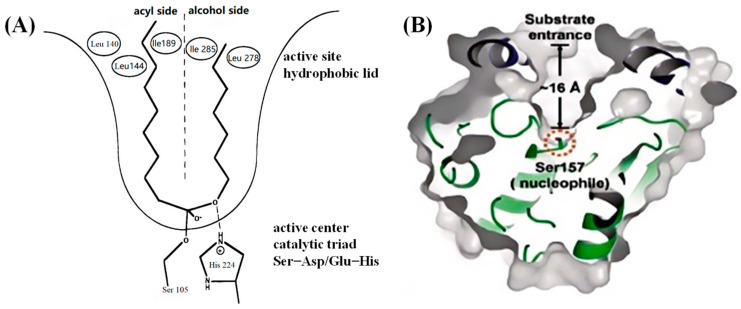
(**A**) Lipase active pocket structure; (**B**) catalytic triplet Ser–Asp–His active center and substrate-specific binding process.

The catalytic mechanism of lipase primarily relies on its unique active site structural characteristics and interactions with substrates (Figure 3). The active center of lipase comprises a serine (Ser) residue, which, in conjunction with aspartic acid (Asp) and histidine (His), forms the catalytic triad. This center is typically covered by an α-helical structure, referred to as the “lid” [34,35]. Upon contact with the oil–water interface, the “lid” undergoes a conformational rearrangement, enhancing its hydrophobicity and thereby exposing the lipase active site, enabling specific binding with the substrate. Through the formation of an acyl–enzyme intermediate while maintaining the stability of the transition intermediate, lipase efficiently catalyzes esterification reactions. This process exemplifies the interfacial activation phenomenon of lipase at the oil–water interface, which constitutes the core of its catalytic mechanism [36].

This phenomenon of interfacial activation not only significantly enhances the affinity of lipases for hydrophobic substrates but also regulates their catalytic activity through dynamic conformational changes, thereby exhibiting unique advantages in heterogeneous systems. Specifically, upon the uncoiling of the lipase “lid” structure at the oil–water interface, the exposure of hydrophobic amino acid residues surrounding the active site not only stabilizes the binding conformation of the substrate with the enzyme but also optimizes the charge relay network composed of Ser–His–Asp by reducing the solvation effect of water molecules on the catalytic triad. This, in turn, accelerates the formation and hydrolysis steps of the acyl–enzyme intermediate. This conformational regulation mechanism stands in stark contrast to the “closed state” of the enzyme in homogeneous solutions. For instance, in organic solvent–water mixed systems, the interfacial activation efficiency of lipases can be enhanced by 2–3 orders of magnitude [37]. Furthermore, molecular dynamics simulations have demonstrated that the displacement amplitude of the “lid” during interfacial activation is positively correlated with the carbon chain length of the substrate, suggesting that it may dynamically adjust the active site cavity size to accommodate fatty acyl chains of varying lengths. This provides a structural basis for lipases to process complex substrates in industrial processes, such as biodiesel synthesis and oil modification [38]. Notably, the “lid” region of some microbial lipases, such as *Candida antarctica* lipase B (CALB), contains flexible loop structures. The conformational fluctuations of these loops may further expand the enzyme’s adaptability to interface curvature, thereby maintaining efficient catalysis even in confined systems, like microemulsions or nanoemulsions [39].

### 3.3. Present Situation of Lipase Used in Synthesis of Ester

Esterification and transesterification reactions are the primary methodologies employed in the enzymatic synthesis of isoamyl acetate. Direct esterification, owing to its high yield and simplicity, is a frequently adopted approach among researchers. In recent years, studies focusing on the enzymatic synthesis of isoamyl acetate have predominantly centered around the utilization of *Candida rugosa* lipase (CRL) as the biocatalyst [40]. Furthermore, there are sporadic reports in the literature concerning the use of other lipases, including *Candida antarctica* lipase (CAL), *Porcine pancreatic* lipase (PPL), *Bacillus licheniformis* lipase (BLL), and *Burkholderia cepacia* lipase (BCL) [41].

During the enzymatic synthesis process, the direct use of free lipase exhibits numerous drawbacks, which limit its application in industrial production [42]. Firstly, the stability of free enzymes is relatively poor, making them susceptible to external factors, such as temperature, pH, organic solvents, and inorganic ions, which can induce enzyme aggregation or degradation, leading to denaturation and inactivation [43]. This instability not only affects the catalytic efficiency of the enzyme but also increases production costs due to the need for frequent enzyme supplementation. Secondly, free enzymes are difficult to separate from the product after the reaction, often becoming impurities in the final product. This not only reduces the purity of the product but also increases the difficulty and cost of subsequent purification [44,45].

**Figure 4:**
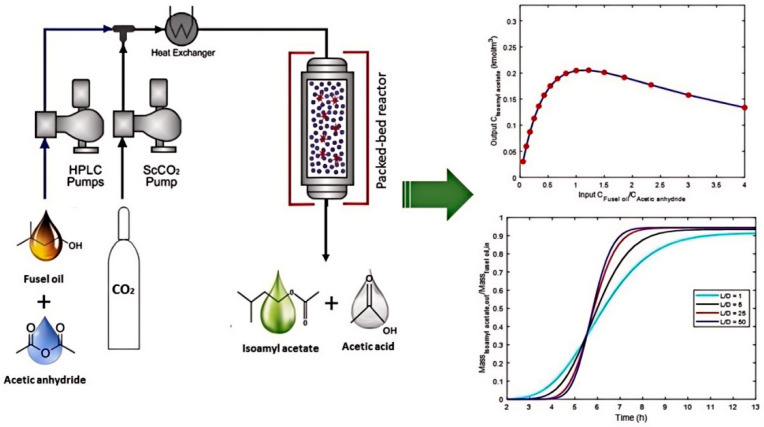
Isoamyl acetate esters were synthesized under supercritical CO_2_ (SC-CO_2_) in a continuous packed-bed reactor (PBR) using immobilized lipase (Novozym 435) as the catalyst [55].

Considering the aforementioned drawbacks, the immobilization of lipase for practical application has emerged as a more effective strategy. By anchoring lipase onto a support material, its stability and reusability can be enhanced, thereby extending the catalyst’s service life. This approach also simplifies the process of product separation and purification, ultimately reducing production costs [46,47]. Furthermore, lipase represents a unique class of interfacial-activated enzymes. Immobilization not only strengthens its adaptability within complex reaction environments but also preserves its catalytic activity [48,49]. Additionally, compared to free enzymes, immobilized enzymes offer the industrial advantage of enabling continuous reactions, which significantly boosts production efficiency [50]. Qian et al. enzymatically synthesized sucrose-6-acetate in organic solvents. Compared with free lipase, the macroporous resin adsorption immobilized lipase-catalyzed sucrose esterification rate increased by 1.7 times [51]. Li et al. immobilized CALB on hydrophobically modified cellulose, resulting in markedly improved stability of the immobilized lipase at pH 9 and 70 °C, with relative activity retentions of 90.90% and 54.80%, respectively [52]. Parneet et al. immobilized CRL on magnetic multi-walled carbon nanotubes, and compared with free lipase, the esterification rate in the final product was increased by 2.3 times [53]. Xia et al. reported an activity recovery rate of 106.18% for *Aspergillus oryzae* lipase (AOL) in their prepared immobilized lipase formulation [54]. Dias et al. utilized Novozym 435 as a catalyst in a continuous packed-bed reactor under supercritical CO_2_ conditions to synthesize isoamyl acetate (Figure 4), ultimately achieving continuous high-yield production (0.21 kmol/m^3^) of isoamyl acetate [55]. These studies have shown that enzyme immobilization is a feasible and powerful means to overcome the defects of free enzyme methods. Therefore, in the process of enzymatic synthesis of isoamyl acetate, the immobilized enzyme technology has significant advantages and can better meet the needs of industrial production.

### 3.4. Immobilization Method of Lipase

The immobilization methods of lipase mainly include physical adsorption, embedding, covalent binding, and crosslinking (Table 1). These four methods have their own advantages and disadvantages. It is necessary to select the appropriate immobilization method according to the characteristics of the enzyme and the catalytic environment.

**Figure 5:**
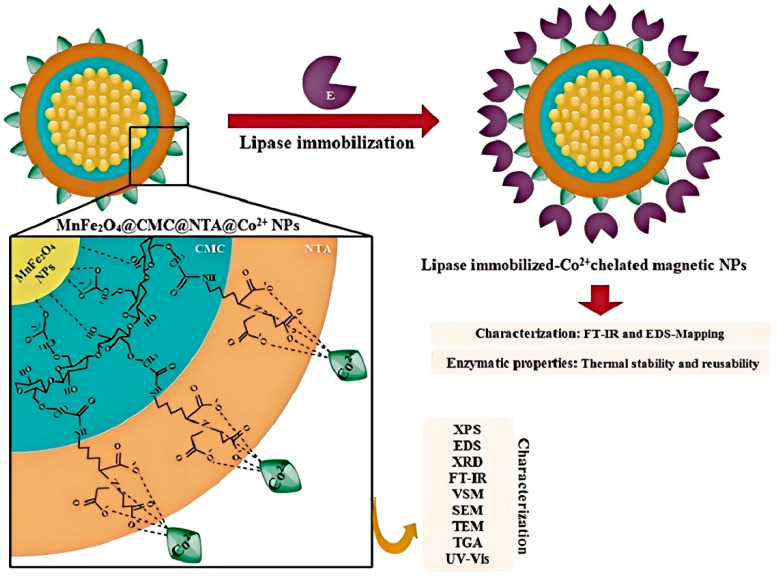
Lipase immobilization was performed via physical adsorption on a novel metal-chelating nanostructure consisting of (S)-N-(5-Amino-1-carboxypentyl) iminodiacetic acid (NTA), as a tetra-dentate chelating agent, grafted onto MnFe_2_O_4_ nanoparticles coated with carboxymethyl cellulose (CMC) for Co^2+^-chelated affinity lipase adsorption [58].

Physical adsorption represents an effective strategy for the direct immobilization of lipase. In this process, lipase adheres to a solid support through physical adsorption forces. The adsorption primarily relies on weak, non-specific interactions, such as van der Waals forces, hydrophobic interactions, hydrogen bonds, and ionic bonds [56]. The main advantages of this method include its simplicity in the immobilization process and the minimal alteration of lipase conformation, which help maintain high lipase activity and stability [57]. Overall, enzyme immobilization via adsorption is a straightforward technique that offers cost-effectiveness while preserving high enzymatic activity (Figure 5) [58]. Compared to other immobilization methods, it involves fewer chemical substances, utilizing only non-toxic, inert supports, which aligns better with food safety requirements [59].

However, this approach also has some drawbacks; for instance, changes in pH, temperature, organic solvents, and ionic strength of the buffer can adversely affect the enzymatic catalytic performance due to the enzyme being predominantly adsorbed on the external surface of the support [60]. Cunha et al. immobilized *Yarrowia lipolytica* lipase on agarose supports via physical adsorption, and the immobilized lipase lost 80% of its activity within 2 h at 50 °C [61]. Additionally, physical adsorption exhibits relatively weak binding forces, which are insufficient to firmly immobilize lipase onto the surface of solid supports. Long-term exposure to the reaction environment can easily lead to lipase leaching [62]. Moreover, the leaching of physically adsorbed lipase may increase under industrial conditions [63]. Mylena et al. immobilized *Pseudomonas cepacia* lipase onto activated carbon (LPAC) via physical adsorption and onto functionalized carbon (LPFC) via covalent immobilization. After a 3 h reaction at 40 °C, the conversion rates of isoamyl acetate were 93.41% and 91.42%, respectively. However, the esterification stability of LPAC upon reuse was inferior to that of LPFC, possibly due to desorption and re-adsorption phenomena during the physical adsorption process [64]. Ghamgui et al. successfully immobilized Staphylococcus simulans lipase (SSL) onto CaCO_3_ via physical adsorption. Under optimal reaction conditions, the yield of isoamyl acetate reached 64%. However, after five cycles of use, a noticeable decline in the yield of isoamyl acetate was observed, attributed to the leaching and loss of lipase from the support material [65].

**Figure 6:**
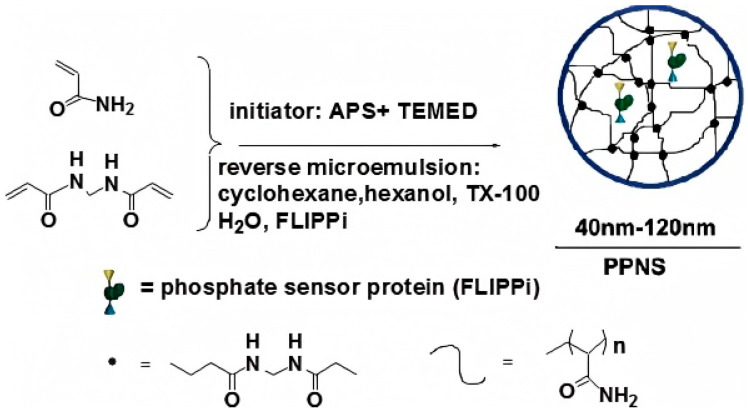
The fluorescent reporter protein (FLIPPi) was embedded into polyacrylamide nanoparticles with a diameter of 40–120 nm [70].

Encapsulation immobilization involves entrapping lipase within a carrier matrix, fibers, lattice structures, or polymer membranes, allowing the simultaneous passage of reactants and products while retaining the lipase [66]. Encapsulation can enhance mechanical stability and reduce lipase leaching [67]. Similar to physical adsorption, this method avoids lipase denaturation caused by chemical methods, as no chemical interactions occur between the encapsulating polymer carrier and the lipase [68]. Polymers, such as alginate, carrageenan, collagen, polyacrylamide, gelatin, silicone rubber, polyurethane, and polyvinyl alcohol containing styryl groups, are commonly used as encapsulating matrices for immobilized enzymes [69]. Sun et al. encapsulated a fluorescent reporter protein (FLIPPi) within polyacrylamide nanoparticles, preserving FLIPPi activity while preventing its degradation by soluble proteases (Figure 6) [70]. Additionally, encapsulation can create an optimal microenvironment for lipase by altering the surface functional groups of different carrier materials. This optimal microenvironment can be achieved using polymers, sol–gels, polymer/sol–gel hybrids, and various inorganic materials [71]. Among these methods, gelation of polyanionic and polycationic polymers through the addition of multivalent counterions is the simplest and most widely applied approach for lipase encapsulation. Zhang et al. successfully prepared dual-embedded activated lipase intrafloral hydrogel microspheres by mixing a lipase in an open conformation co-crystallized with Ca_3_(PO_4_)_2_ with a sodium alginate solution, utilizing Ca^2+^ as the gelating polyvalent counterion. The immobilized lipase exhibited improved thermal and pH stability, with an enzyme activity retention rate of 83% even after 10 cycles of reuse [72].

However, the embedding method has notable drawbacks. As the reaction progresses, the aging of the carrier polymer leads to larger matrix pores, facilitating the leaching of lipase from the carrier [73]. Additionally, unlike physical adsorption, enzymes often participate in polymerization reactions during the embedding immobilization process, which can adversely affect enzyme activity. Achieving uniform pore size in polymeric carrier materials during immobilization is challenging, ultimately resulting in mass transfer difficulties for substrates within the immobilized enzyme during catalysis [74,75]. Lee et al. combined silica sol–gel with multi-walled carbon nanotubes for the embedding immobilization of CRL. In their study, immobilized lipase without the addition of multi-walled carbon nanotubes exhibited complete loss of activity after five reuse cycles, attributed to rapid deactivation caused by lipase leaching into the reaction medium [76]. Kanwar et al. employed a simple embedding technique to immobilize Pseudomonas lipase in a polyvinyl alcohol (PVA) membrane for the synthesis of isoamyl acetate via transesterification reactions [77]. After 3 months of storage at room temperature, the immobilized lipase retained 62% higher activity compared to the free lipase. However, the maximum reaction rate (V_max_) of the immobilized lipase was only 41.86% of that of the free enzyme, owing to mass transfer resistance imposed by the PVA membrane embedding.

Covalent binding is an irreversible enzyme immobilization process primarily achieved through the interaction between amino acid residues on side chains and carrier materials. These amino acid residues typically include amino groups (lysine), thiol groups (cysteine), and carboxyl groups (aspartic acid and glutamic acid), as well as phenol and imidazole groups that are not required for the catalytic activity of lipases [78]. The effectiveness of covalent binding between lipases and carrier materials depends on the shape, surface, size, and chemical structure of the carrier materials, such as agarose, cellulose, polyvinyl chloride, and porous glass [79]. During the covalent binding process, lipases undergo chemical reactions with the active groups on the surface of carrier materials, which is a critical factor determining the stability of the biocatalyst. These active groups on the carrier material surface can either be naturally present in the chemical structure of the carrier or introduced through post-modification.

**Figure 7:**
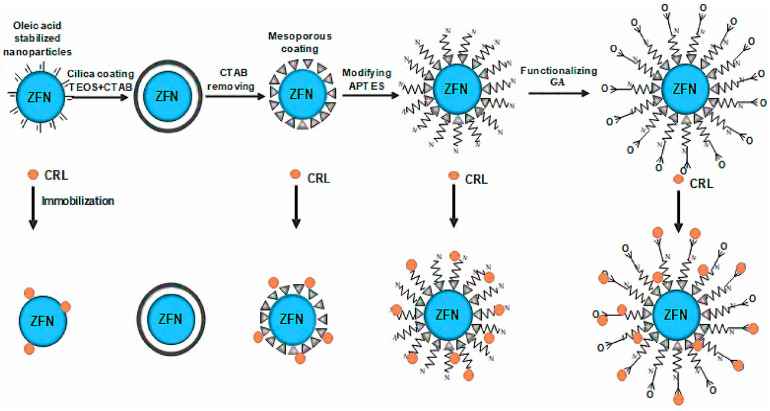
Mesoporous zinc ferrite nanoparticles coated with an amine-functionalized mesoporous silica structure (ZnFe_2_O_4_@MS) were synthesized via the solvothermal method [39].

Compared to other immobilization strategies, covalently immobilized enzymes exhibit superior reusability, stability, and reduced lipase leaching [80,81,82]. Hosseinzadeh et al. successfully immobilized CRL in mesoporous zinc ferrite nanoparticles by covalent bonding (Figure 7) [39]. The activity of the immobilized enzyme was 2.13 times that of the free enzyme, and the thermal stability of the immobilized enzyme and the antibacterial activity against *Staphylococcus aureus* were significantly enhanced. Finally, the yield of isoamyl acetate was 64% by catalyzing the esterification reaction of acetic acid and isoamyl alcohol in n-hexane solvent at 45 °C for 4 h. However, in the process of covalent binding, due to the need for enzymes to participate in chemical reactions, the spatial conformation of enzymes is easy to change, resulting in a decrease in enzyme activity. Bayramoglu et al. covalently immobilized CRL on polydopamine-grafted magnetic chitosan microspheres, which improved the thermal stability and storage stability of the enzyme [38]. However, with the increase in the polydopamine grafting degree, the activity of the immobilized enzyme was only 60–80% of that of the free enzyme.

Crosslinking is achieved through intermolecular crosslinking between lipase molecules and di- or multifunctional compounds [83]. In this process, carrier materials may not be employed, and this technique is also referred to as carrier-free immobilization [84]. Under such circumstances, lipase can act as its own support, yielding nearly pure lipase while eliminating both the advantages and disadvantages associated with carrier materials. Crosslinking methods can overcome the drawback of low spatial utilization of enzyme proteins caused by the use of carrier materials [85]. In recent years, glutaraldehyde has been predominantly used as a protein crosslinker due to its low cost and availability, facilitating polymerization reactions through the reaction of free amino groups in enzyme proteins with glutaraldehyde [86].

**Figure 8:**
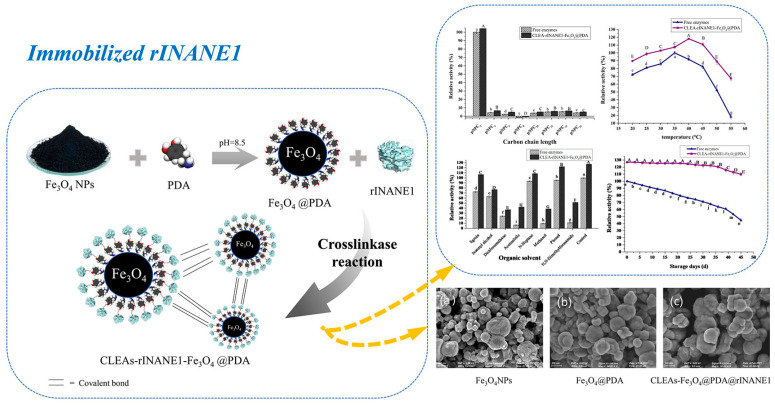
rINANE1 was successfully immobilized on polydopamine (PDA)-modified magnetic ferric oxide nanoparticles (Fe_3_O_4_NPs) by adsorption–precipitation–crosslinking to obtain the crosslinked enzyme aggregate (CLEA)–rINANE1–Fe_3_O_4_@PDA. The SEM diagram of Fe_3_O_4_ (**a**), Fe_3_O_4_@PDA (**b**) and CLEA–rINANE1–Fe_3_O_4_@PDA (**c**). The means with different letters showed significant differences (*p* < 0.05) [87].

Immobilized enzymes prepared via carrier-free immobilization methods typically exhibit small particle sizes and low mechanical strength, rendering them suitable only for enzymes with small molecular substrates and products, and not for reactions involving large molecules that require vigorous stirring. To expand the applicability of crosslinking methods, scientists often combine them with carrier materials. Luo et al. utilized pectin as a crosslinker to prepare magnetic crosslinked enzyme aggregates, which retained 82.72% relative activity after eight usage cycles and exhibited negligible changes in enzyme activity after 45 days of storage, demonstrating excellent reusability and storage stability (Figure 8) [87]. Sóti et al. employed poly(glycerol methacrylate) as a crosslinker to immobilize CALB onto poly(vinyl alcohol) and poly(lactic acid) membrane nanofiber carriers, with the resulting immobilized lipase retaining over 80% of its biocatalytic activity after ten cycles of use [88].

### 3.5. Selection of New Immobilized Lipase Carrier

Currently, the performance of immobilized enzymes is primarily determined by two aspects, namely the immobilization method and the selection of the immobilization carrier. The adoption of an appropriate immobilization method and the selection of an excellent immobilization carrier are particularly crucial for enhancing the activity and stability of immobilized lipase [89].

A good carrier material should possess properties, such as thermal and chemical stability, renewability, and low cost [90]. Additionally, the structure and surface of the carrier should be easily modulated. The lid structure of lipase can be opened by functional groups on the carrier surface, facilitating the binding of the enzyme active site to the substrate molecule and, thus, enhancing enzyme activity. Meanwhile, by regulating the carrier structure, substrate diffusion limitations can be reduced, accelerating the enzymatic catalytic reaction process.

One of the critical issues that must be addressed is the removal of the catalyst from the reaction system after the completion of the biocatalytic process. A favorable approach to overcome this challenge involves immobilizing lipase molecules onto magnetic materials, enabling the facile separation of the immobilized lipase using an external magnet or magnetic field [91]. In addition to the presence of numerous hydroxyl groups, magnetic nanoparticles are also renowned for their large surface area, which facilitates easy modification and robustness when covalently bound to lipase. Magnetic materials exhibit significant advantages, such as excellent mechanical stability and low porosity, which minimize steric hindrance and foster the formation of a stable enzymatic biocatalytic matrix [92].

**Figure 9:**
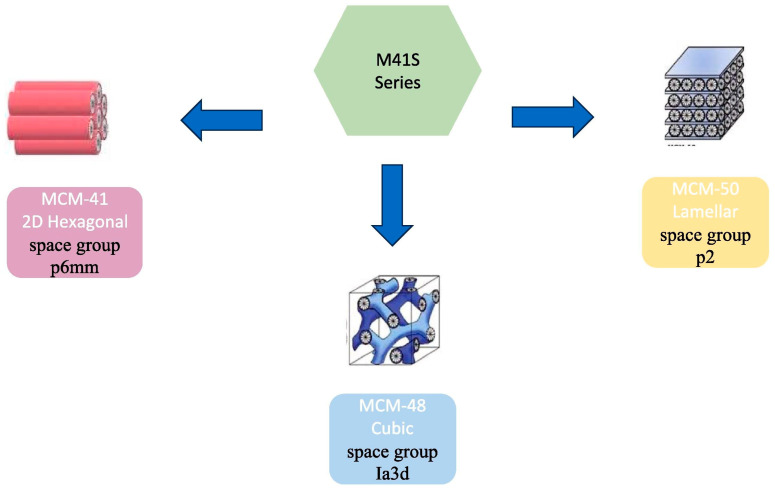
Structural diversity in the M41S series of mesoporous silica materials [95].

The primary characteristic of mesoporous materials lies in their ability to obtain the desired porous matrix by adjusting synthesis parameters, enabling the stable attachment of lipase, which is also the key distinguishing feature from other lipase immobilization materials [93]. These carriers comprise ordered mesopores with pore sizes ranging from 2 to 50 nm, a density of approximately 1 g/cm^3^, and a specific surface area exceeding 1500 m^2^/g, making them excellent immobilization carriers for various lipases and other biomolecules [94]. Due to their insolubility in water, hydrophilicity, sufficient active groups, and chemical and thermal stability, mesoporous materials meet many requirements for lipase immobilization carriers (Figure 9) [95]. During lipase immobilization on mesoporous surfaces, adsorption and covalent binding are typically employed [96]. Common mesoporous materials include SBA-15, zeolites, and ordered mesoporous structured oxides [97].

**Figure 10:**
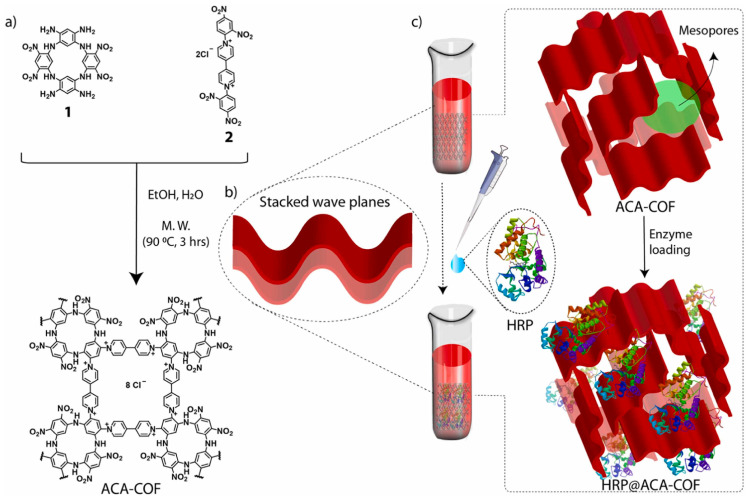
Horseradish peroxidase (HRP) was immobilized on a cationic macrocycle-based covalent organic framework (COF) by physical adsorption. (**a**) Reaction scheme for the synthesis of ACA-COF. (**b**) Schematic representation of layer stacking in ACA-COF. (**c**) Schematic representation of the physical immobilization of HRP in ACA-COF [99].

Covalent organic frameworks (COFs) represent a novel class of non-metallic porous materials that exhibit distinct advantages over conventional support materials, such as a high specific surface area, excellent thermal stability, and tunable structures [98]. Currently, physical adsorption (Figure 10) [99] or covalent bonding [100] methods are commonly employed for enzyme immobilization using COFs. However, due to the limited pore size of COFs, enzymes tend to be adsorbed or covalently linked to the external surface of COFs, which often diminishes the operational stability of immobilized lipases. In recent years, numerous researchers have achieved in situ encapsulation of enzymes within COF materials, yielding immobilized enzymes with high enzyme loading and reduced leakage.

### 3.6. Research Progress on Synthesis of Isoamyl Acetate Catalyzed by Immobilized Lipase

A comprehensive review of the literature published in the past decade on the immobilized lipase-catalyzed synthesis of isoamyl acetate was conducted, with the results summarized in Table 2. Among the studies focusing on the immobilized enzyme-catalyzed synthesis of isoamyl acetate, CRL emerged as the most frequently employed catalyst, primarily due to its generally high efficiency in catalyzing esterification reactions, with yields typically exceeding 85%. Approximately half of the relevant research utilized physical adsorption as the immobilization method, owing to its operational simplicity and the ability to largely preserve lipase activity. The direct esterification method utilizing acetic acid and isoamyl alcohol for the synthesis of isoamyl acetate accounts for 70% of the relevant studies. Following immobilization, lipases exhibited a slight elevation in their optimal catalytic reaction temperatures, predominantly ranging from 40 to 50 °C, reflecting the enhanced operational stability conferred by enzyme immobilization. Moreover, the elevated optimal temperatures facilitated accelerated reaction rates and reduced reaction times, thereby offering significant potential for the industrial-scale production of isoamyl acetate.

Zhang et al. employed CRL and consistently opted for the simplest direct esterification approach to synthesize isoamyl acetate. Initially, starting from the enzyme immobilization strategy, a dual immobilization method combining physical adsorption and hydrophobic gel entrapment was utilized to immobilize the lipase. Under reaction conditions of 50 °C for 8 h, the yield of isoamyl acetate reached as high as 85.19% [1]. Subsequently, the lipase was encapsulated in situ within a COF under aqueous conditions, achieving an even higher yield of 86.94% within a shorter reaction time of 7 h at 50 °C [5]. The observation of elevated isoamyl acetate yields under reduced reaction durations underscores the pivotal role of a well-designed immobilization strategy in facilitating optimal lipase activity expression. Finally, the COF-encapsulated lipase was deployed in a deep eutectic solvent (DES), resulting in a further enhanced yield of 98.26% for isoamyl acetate after 7 h of reaction at 50 °C [6]. This outcome highlights the significant impact of the reaction microenvironment provided by an optimized reaction medium, demonstrating how solvent engineering can effectively amplify enzymatic catalysis and profoundly influence reaction kinetics.

## 4. Prospect of Solvent Engineering to Enhance the Enzyme Catalytic Process

Enzyme catalytic process enhancement technologies can be categorized into physical and chemical enhancements. Physical enhancements primarily include ultrasonic disruption, microwave irradiation, and continuous flow reactors. Chemical enhancements mainly encompass organic solvents, metal ions, surfactants, and other compounds. Currently, solvent engineering for enhancing enzyme catalytic processes has garnered significant attention from researchers due to its advantages of simplicity in operation and high controllability.

**Figure 11:**
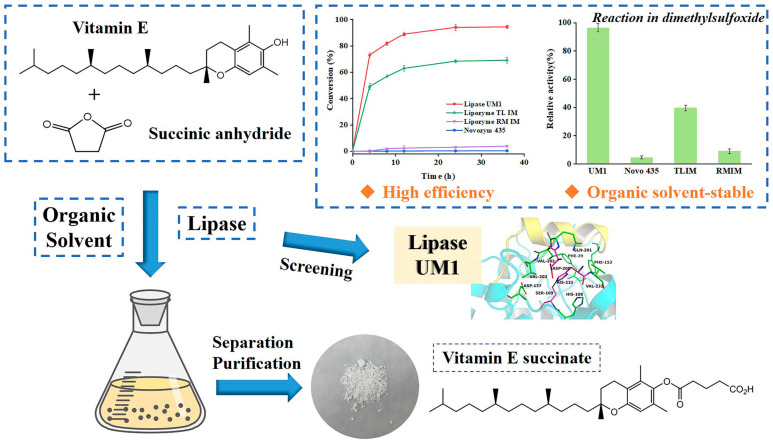
Vitamin E succinate (VES) was synthesized through the catalysis of the organic solvent-stable lipase UM1, resulting in an exceptional conversion [113].

The addition of an appropriate amount of organic solvent to the reaction system can reduce the viscosity of the system, thereby decreasing mass transfer resistance. The reduction in mass transfer resistance facilitates the diffusion and transport of substrates and products within the reaction system, enabling more thorough contact between enzymes and substrates, and consequently enhancing the rate of enzymatic reactions. The selection of a suitable organic solvent can activate enzyme molecules to a certain extent, thereby increasing their catalytic activity (Figure 11) [113]. This may be attributed to the interaction between the organic solvent and the enzyme molecule, which alters the enzyme’s conformation, making it more conducive to substrate binding and catalysis. Additionally, the organic solvent may form stable interactions with the enzyme molecule, thereby protecting it from damage by the external environment. The presence of organic solvents can also mitigate side reactions predominantly driven by water. Enayati et al. employed CALB to enzymatically synthesize lactyl fatty acid esters using fatty acids and lactose as substrates in an organic solvent [114]. The results indicated that n-hexane and acetonitrile yielded the highest substrate conversion rates for both free and immobilized lipase, achieving rates of 77% and 93%, respectively. Furthermore, it was noted that the esterification conversion rate of free lipase was solvent-dependent, whereas the conversion rate of immobilized lipase exhibited a lower degree of dependency on the solvent.

**Figure 12:**
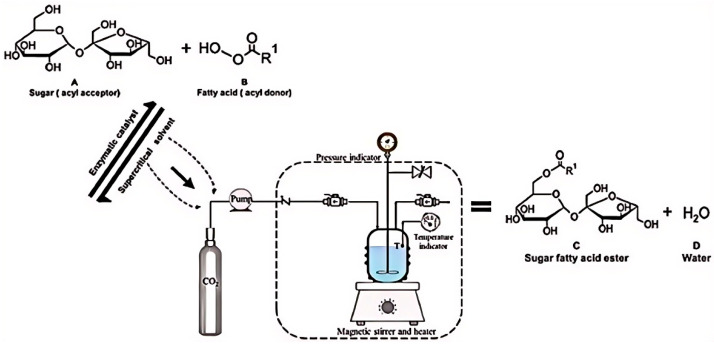
A usual high-pressure device applied to enzymatic sugar esters synthesis at supercritical conditions [117].

Supercritical fluids represent a unique state of matter where substances exist at temperatures and pressures exceeding their critical values, exhibiting both the high-density solubility of liquids and the low-viscosity diffusivity of gases [115]. As green media for enzymatic catalytic reactions, supercritical fluids can significantly influence enzyme molecular conformations and substrate diffusion efficiencies by modulating their density and solvation capabilities. Their low surface tension facilitates the dissolution of hydrophobic substrates, thereby mitigating mass transfer resistance, while their temperature sensitivity simplifies product separation and enzyme recovery [116]. Studies have demonstrated that supercritical fluid media can enhance the catalytic activities of enzymes, such as lipases and proteases, accelerating reaction processes, such as transesterification and hydrolysis, while maintaining the three-dimensional structural stability of enzymes (Figure 12) [117]. Lee et al. investigated the kinetic models of mixed immobilized lipase (MIL)- and co-immobilized lipase (CIL) systems based on the transesterification reaction of soybean oil and methanol, revealing that the initial reaction rates of MIL and CIL increase under supercritical fluid conditions when the methanol concentration does not exceed twice that of the oil [118].

**Figure 13:**
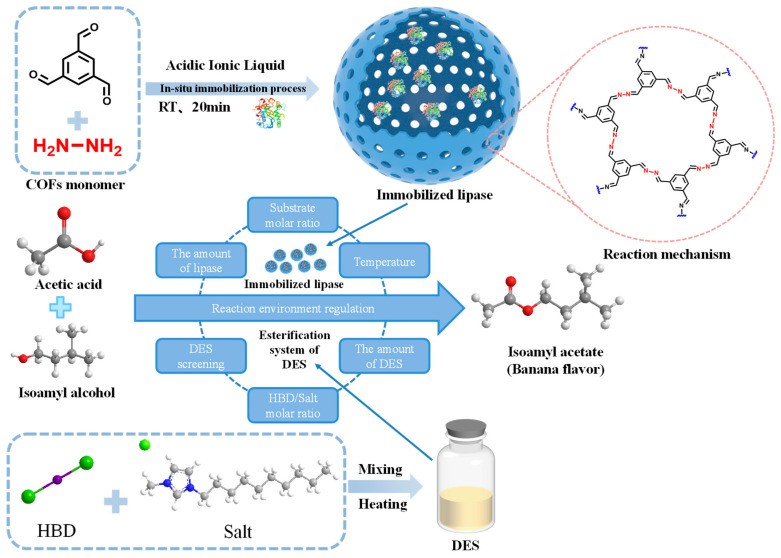
CRL was immobilized in COF carriers by in situ encapsulation in an aqueous phase at room temperature. Furthermore, the above-prepared immobilized lipase was applied to catalyze the synthesis of isoamyl acetate in different deep eutectic solvent (DES) environments [6].

Over the past several decades, the substitution of hazardous volatile organic solvents with novel eco-friendly solvents boasting health and safety attributes has garnered significant attention. Among these, ionic liquids (ILs) and deep eutectic solvents (DESs) have attracted considerable interest due to their exceedingly low vapor pressures, high chemical and thermal stabilities, and capacity to readily dissolve a diverse range of compounds [119,120]. Liu et al. immobilized *Yarrowia lipolytica* lipase onto magnetic iron oxide supports and catalyzed the efficient kinetic resolution of (R, S)-2-octanol in mixed ionic liquids. The catalytic performance of the immobilized lipase did not exhibit a notable decline even after five reaction cycles in the mixed ionic liquid medium [121]. Papadopoulou et al. opted for ILs and DESs as media for lipase-inorganic hybrid nanoflower-catalyzed hydrolysis and synthesis reactions [122]. An extremely low reaction yield was observed in all cases studied when hydroxyl ammonium-type ionic liquids were employed as the reaction medium. This phenomenon was attributed to the high capability of most water-soluble ionic liquids to strip tightly bound water from protein molecules, thereby causing lipases to lose their biocatalytic activity under low water content conditions [123,124]. Additionally, they utilized ethylene glycol (EG) as a hydrogen bond donor to prepare DESs as solvents for the CALB nanoflower-catalyzed hydrolysis of p-nitrophenyl butyrate (p-NPB). Under these conditions, the hydrolytic activity of CALB nanoflowers was twice as high as that observed in buffer solutions. This enhancement was attributed to the lower viscosity of EG-based DESs [125], which reduced mass transfer limitations and thereby increased the biocatalytic activity of the immobilized enzyme. Furthermore, the residual activity of CALB nanoflowers after 24 h of incubation in a system with choline chloride as the hydrogen bond acceptor was twice that observed in buffer solutions. The stabilizing effect of DESs can be ascribed to specific interactions between the solvent and protein molecules, which may lead to a more rigid and stable enzyme structure (Figure 13) [6,126]. Given their superior solvent properties and high tunability, DESs hold significant potential as green media in biocatalytic processes.

## 5. Conclusions

This review article summarizes the current research status of immobilized lipases in the catalytic synthesis of isoamyl acetate, highlighting the pivotal role of immobilization techniques in enhancing the stability, reusability, and catalytic efficiency of lipases. Despite significant advancements in immobilization methods, carrier selection, and the elucidation of enzymatic catalytic mechanisms, particularly the remarkable catalytic performance demonstrated by specific lipases, such as Candida rugosa, lipase after immobilization, which renders them suitable for large-scale production of isoamyl acetate, several limitations persist. These include the potential reduction in enzyme activity associated with certain immobilization methods, the high cost of some novel carrier materials, and technological bottlenecks hindering industrial applications. Future research endeavors should focus on exploring cost-effective, high-performance immobilization carrier materials, optimizing immobilization processes to preserve maximal enzyme activity, and enhancing the application of solvent engineering in enzymatic catalysis, with a particular emphasis on investigating catalytic efficiency in green solvents and supercritical fluid media. Moreover, integrating modern biotechnology and materials science to develop novel immobilization technologies is crucial for improving the stability and catalytic efficiency of lipases. This will address the urgent demand for natural, high-efficiency food additives in the food industry and facilitate the industrial production of flavor esters, such as isoamyl acetate.

## Figures and Tables

**Table 1:** Different immobilization methods with enzymes.

Classification	Adsorption	Embedding	Covalent Bonding	Crosslinking
**Advantages**	Simple method, little loss of activity, cheap and fast	Large amount of immobilized enzyme, no need for extraction or purification, low loss of activity	Strong bonding properties, excellent stability	Strongly binds to lipase, good stability in aqueous solution
**Disadvantages**	Leaks easily, binds non-specifically	Methodological complexity, mass transfer limitations, leakage	Increased cost, decreased activity	May be inactive, lack of mechanical properties, difficult to control size

**Table 2:** Immobilized lipase-catalyzed synthesis of isoamyl acetate.

Lipase	Carrier (Immobilization Method)	Reaction Method	Temperature (°C)	Time (h)	Yield (%)	Reference
*Candida rugosa* lipase	COFs (embedding)	Esterification	50	7	86.94	[5]
*Candida rugosa* lipase	SBA-15, calcium alginate gel (adsorption, embedding)	Esterification	50	8	85.19	[1]
*Candida rugosa* lipase	Magnetic chitosan beads (covalent bonding)	Esterification	35	24	98.4	[38]
*Candida rugosa* lipase	COFs (embedding)	Esterification	50	7	98.26	[6]
*Candida rugosa* lipase	Epoxy-activated cloisite 30B (covalent bonding)	Esterification	50	4	91.6	[101]
*Candida rugosa* lipase	ZnFe_2_O_4_@MS (covalent bonding)	Esterification	45	4	64	[39]
*Candida rugosa* lipase	PDA@Co-MWCNT (covalent bonding)	Esterification	45	24	75	[102]
*Porcine pancreatic* lipase	ILs/MZIF-90 (adsorption)	Esterification	45	9	85.5	[103]
*Porcine pancreatic* lipase	ILs/Fe_3_O_4_@MOF (covalent bonding)	Esterification	45	24	75.2	[104]
*Porcine pancreas* lipase	Activated carbon (adsorption)	Esterification	40	3	93	[64]
*Porcine pancreas* lipase	Metallized activated carbon (adsorption)	Esterification	40	4	96.62	[105]
*Pseudomonas fluorescens* lipase	Octyl-silica (adsorption)	Esterification	37	24	96.9	[106]
*Pseudomonas fluorescens* lipase	Mesoporous silica matrix (adsorption)	Transesterification	40	20	62	[107]
*Thermomyces lanuginosus* lipase	K_2_SO_4_ crystal (adsorption)	Transesterification	50	6	95	[41]
*Thermomyces lanuginosus* lipase	Fe_3_O_4_ (adsorption)	Transesterification	30	7	61	[108]
*Rhizomucor miehei* lipase	Al_2_O_3_-NP (adsorption)	Transesterification	50	1	15.4	[109]
*Candida antarctica* lipase B	Purolite^®^ MN102 (adsorption)	Transesterification	75	6	100	[110]
*Aspergillus oryzae* lipase	Calcium alginate gel (embedding)	Transesterification	68.5	6	89.55	[111]
*Bacillus aerius* lipase	Silica gel matrix (covalent bonding)	Esterification	55	10	68.38	[26]
*Burkholderia cepacian* lipase	Calcium alginate gel (embedding)	Esterification	37	120	92	[112]

## Data Availability

No new data were created or analyzed in this study.
